# Spatiotemporal variations of ecosystem services in the Aral Sea basin under different CMIP6 projections

**DOI:** 10.1038/s41598-024-62802-9

**Published:** 2024-05-28

**Authors:** Jing He, Yang Yu, Lingxiao Sun, Chunlan Li, Haiyan Zhang, Ireneusz Malik, Malgorzata Wistuba, Ruide Yu

**Affiliations:** 1 State Key Laboratory of Desert and Oasis Ecology, Xinjiang Institute of Ecology and Geography, Chinese Academy of Sciences, Urumqi, 830011 China; 2 Cele National Station of Observation and Research for Desert-Grassland Ecosystems, Cele, 848300 China; 3 School of Environment and Material Engineering, Yantai University, Yantai, 264005 China; 4 Polish-Chinese Centre for Environmental Research, Institute of Earth Sciences, University of Silesia in Katowice, Bankowa 12, 40-007 Katowice, Poland

**Keywords:** Aral Sea basin, Ecosystem services, Scenario simulation, InVEST model, Trade-off and synergy, Ecosystem services, Environmental impact

## Abstract

The Aral Sea, located in Central Asia, has undergone significant reduction in surface area owing to the combined impacts of climate change and human activities. This reduction has led to a regional ecological crisis and profound repercussions on ecosystem services. Investigating the spatiotemporal variations and synergistic trade-offs of ESs in the Aral Sea basin is crucial for fostering the integrated development of the region’s socioeconomic ecology. This study utilizes the Future Land-Use Simulation and InVEST models to analyze future land-use scenarios, integrating CMIP6 projections to assess the quality of four key ecosystem services: water production, soil conservation, carbon storage, and habitat quality over two timeframes: the historical period (1995–2020) and the projected future (2021–2100). Employing Spearman correlation, the study explores the trade-offs and synergies among these ecosystem services. Findings reveal that the primary forms of land-use change in the Aral Sea basin are the reduction in water area (− 49.59%) and the rapid expansion of urban areas (+ 504.65%). Temporally, habitat quality exhibits a declining trend, while carbon storage shows an increasing trend, and water production and soil retention fluctuate initially decreasing and then increasing. Spatially, water production and carbon storage demonstrate an increasing trend from the northwest to the southeast. Habitat quality exhibits a higher spatial pattern in the southeast and south, contrasting with lower spatial patterns in the north and west. Low-level soil conservation is predominantly distributed in the northwest, while medium to low-level soil conservation is prevalent in the east of the basin. The trade-off and synergy analysis indicates that between 1995 and 2020, a trade-off relationship existed between carbon storage and habitat quality and water production, whereas synergies were observed between soil conservation and carbon storage, water production and habitat quality, and soil conservation. The correlation between water production and soil conservation emerges as the strongest, whereas the correlation between carbon storage and habitat quality appears to be the weakest. The dynamic spatiotemporal changes, trade-offs, and collaborative relationships of ESs constitute major aspects of ecosystem service research, holding substantial implications for the effective management of the regional ecological environment.

## Introduction

Ecosystem services (ESs) are fundamental benefits provided by natural ecosystems to humanity, serving as a critical link between natural ecosystems and human society^1^. The key ESs influencing arid regions include water yield (WY), soil conservation (SC), carbon storage (CS), and habitat quality (HQ). Central Asia, home to one of the largest arid expanses globally, features the Aral Sea basin as a quintessential example within the realm of Central Asian basins.

Over an extensive temporal trajectory, nations inhabiting the Aral Sea basin have exploited water resources without stringent constraints in their pursuit of economic advancement^2^. This unregulated appropriation, coupled with the unsustainable exploitation of water resources and the lack of effective regulatory mechanisms^3^, has led to a significant reduction in water levels and the consequent shrinkage of the Aral Sea’s area^4–6^.

Limited water resources restrict vegetation growth and carbon sequestration. Vegetation growth influences soil retention through processes such as evapotranspiration and concurrent water sequestration during carbon sequestration. Soil quality plays a crucial role in sustaining biodiversity, productivity, and structural support for plant life and other organisms. These ESs exhibit complex interplay and interconnectedness, as evidenced by prior research^7,8^. Huang et al. (2022) found that the desiccation of the Aral Sea results not only from agricultural expansion but is also exacerbated by climate change and increased actual evapotranspiration. This underscores the urgency of addressing the consequences of the sea’s contraction on human well-being and economic development. It is imperative to predict future changes in ESs and comprehend their interconnections across the upper, middle, and lower regions of the Aral Sea basins.

Research efforts have underscored the predictive capabilities of integrated models like the FLUS and InVEST models in anticipating future land-use patterns and the allocation of ESs^9^. employed a coupled approach combining FLUS and InVEST to simulate terrestrial ecosystem carbon stocks in China up to 2100, based on the Representative Concentration Pathways (RCPs). Regarding climate change projections, the emerging International Coupled Model Intercomparison Project Phase 6 (CMIP6) enhances global climate data by harmonizing shared socioeconomic pathways (SSPs) and RCPs, leading to more robust and reliable predictions^10^. A crucial component within CMIP6 is the Scenario Model Intercomparison Project, which integrates SSPs and RCPs to advance a scientifically refined combination of socioeconomic and radiative forcing scenarios. This project presents multiple radiative forcing pathways and introduces five shared socioeconomic pathways (SSP1-5), along with seven canonical concentration pathways. A comprehensive matrix of these pathways is constructed, drawing from seven SSP scenarios and includes two benchmark scenarios for comparison^11,12^.

Existing research, such as that by Li et al. (2021a), has utilized prospective simulation data from CMIP6, employing four distinct climate models from the CMIP6 ensemble to analyze the intricate interplay and potential synergies among critical ESs in the Central Asia region. This analysis explores the complex relationship between forthcoming climate variations and socioeconomic progress. In a related study, Zhu et al.^13^ conducted a meticulous assessment of modeling proficiency for China, employing a cohort of 12 CMIP6 global climate models. Furthermore, Lu et al.^14^ meticulously examined five global climate models from CMIP6, exploring prospective shifts in climate conditions across seven integrated scenarios encompassing both SSPs and RCPs.

However, most studies investigating ESs in future scenarios have typically focused on a single service, leading to a limited understanding of the interconnectedness among various services within the context of diverse climate and economic change scenarios. This study primarily seeks to evaluate how trade-offs and synergies among multiple ESs manifest within various developmental trajectories.

To achieve this aim, we employ the following methodologies. Our analysis is anchored in an innovative combination of RCP and SSP frameworks. We simulated prospective land-use patterns spanning from 2021 to 2100, considering four distinct radiation forcing pathways alongside four alternative policy scenarios (SSP1, SSP2, SSP3, and SSP5). Our investigation looks into anticipated alterations in WY, SC, CS, and HQ across the SSP126, SSP245, SSP370, and SSP585 scenarios. We elucidate the differences in trade-offs and synergies among ESs within the Aral Sea basin. Basing on these findings, we provide suggestions for land-use planning and propose ecological management measures designed to enhance the sustainability of ESs. These contributions carry practical importance and hold considerable scientific value in the ongoing effort to understand the causes of the Aral Sea Crisis and identify viable solutions.

## Materials and methods

### Study area

**Figure 1:**
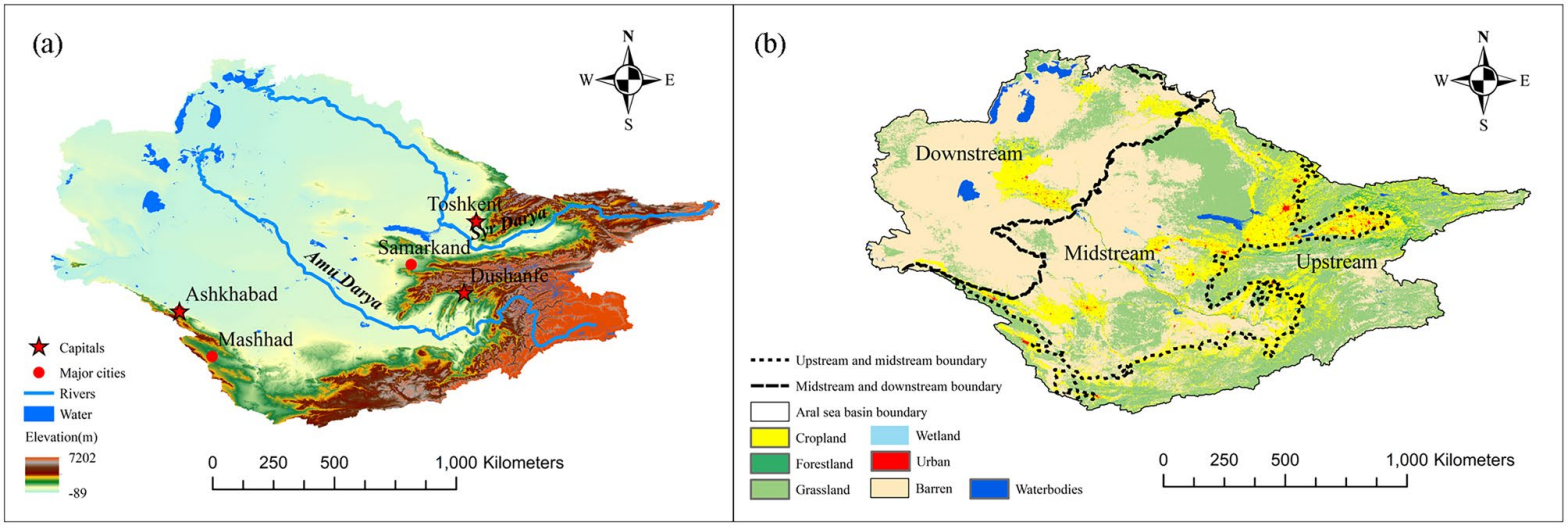
Map showing the location and spatial pattern of elevation (**a**) and distribution of Land use (**b**) in the Aral sea basin.

The Amu Darya River and Syr Darya River are two prominent river systems within the Aral Sea basin, situated in central Eurasia with a total size of approximately 1.7 × 10^6^ square kilometres. According to Yu et al. (2019), the upstream portions of the basin are comprised of Tajikistan and Kyrgyzstan, whilst the downstream regions consist of Uzbekistan, Turkmenistan, and Kazakhstan. The topography of the region displays variations in elevation, with higher elevations found in the eastern part and lower elevations in the western part. This topographical diversity is influenced by the presence of the Tianshan Mountains, Pamir Plateau, Karakum Desert, and Kyzylkum Desert. The natural geography of the region includes deserts, oases, mountains in the southern area, and grasslands, plains, and hills in the northern area, which contribute to a wide range of geomorphological features (Fig. 1). The climate in the region is classified as temperate continental, as described by Yu et al. (2020), with an average temperature of approximately 11 °C. The annual precipitation in the region is estimated to be around 236 mm, while the evaporation rate can reach up to 1700 mm, as reported by Conrad et al., 2012.

### Data sets

#### Climate data

The present analysis utilizes historical climate data obtained from the high-resolution grid dataset provided by the Climate Research Unit (CRU) at the University of East Anglia. The dataset covers the timeframe from 1995 to 2020 and employs the CRU TS v4.03 dataset, accessible at the following URL: https://crudata.uea.ac.uk/cru/data/hrg/. This study utilizes data from four well-known global climate models, namely CanESM5, IPSL-CM6A-LR, MIROC6, and MRI-ESM2-0, to develop future climate forecasts for the period from 2021 to 2100. Specific details of these models can be found in Table 1. CMIP6 encompasses a wide range of climate model versions from 33 universities worldwide^10^.

**Table 1:** List of climate models provided by the CMIP6 in this study.

Model name	Longitude × latitude	Period	Institute	Resolution (lon × Lat, L: vertical layer)
CanESM5	2.8125° × 2.7906°	2021–2040, 2041–2060, 2061–2080, 2081–2100	Canadian Centre for Climate Modeling and Analysis (CCCMA), Canada	128 × 64, L49
IPSL-CM6ALR	2.5° × 1.2676°		Institute Pierre Simon Laplace (IPSL), France	144 × 143, L79
MIROC6	1.4063° × 1.4008°		Japan Agency for Marine-Earth Science and Technology (JAMSTEC), Japan	256 × 128, L81
MRI-ESM2-0	1.125° × 1.1215°		Meteorological Research Institute (MRI), Japan	320 × 160, L80

The present investigation aims to elucidate long-term patterns from 2021 to 2100 by integrating models containing both SSPs and RCPs). Four situations, namely, SSP126, SSP245, SSP370, and SSP585, have been intentionally selected in accordance with the parameters of the study, as outlined in Table 2. The scenario labeled as SSP126 embodies the “Green Path for Sustainability,” characterized by minimal challenges in both mitigation and adaptation, alongside lower greenhouse gas emission levels. Furthermore, SSP126 aligns with the coercive force of (SSP1)-RCP2.6. Conversely, SSP370 embodies a more pessimistic outlook on forthcoming economic and social development, characterized by constrained investments in education and health in impoverished nations. This predicament is compounded by rapid population growth and escalating inequality, culminating in significant challenges for mitigation and adaptation endeavors. Moreover, its (SSP3)-RCP7.0 configuration is situated within the domain of medium to higher radiative forcing scenarios. In parallel, SSP585 envisions a world characterized by swift fossil fuel advancement, wherein (SSP5)-RCP8.5 propels substantial levels of greenhouse gas emissions. These scenarios have been documented by^15,16^.

**Table 2:** Datasets used in this study.

Data	Year	Resolution	Resource
Climate	1995–2015	0.5°	https://crudata.uea.ac.uk/cru/data/hrg/
	2021–2100	1 km	WorldClim v2.1
Soil	–	1 km	HWSD, Harmonized World Soil Database v1.2
Land use	1995–2015	300 m	European Space Agency climate change initiative
	2021–2100	0.25°	Land-Use Harmonization
Elevation	–	90 m	http://www2.jpl.nasa.gov/srtm/
Population	2015	1 km	https://www.worldpop.org/
Road network	2015	1 km	http://www.openstreetmap.org/

To adhere to the paper’s specifications, we initially applied bias correction to the outputs of all General Circulation Models (GCMs), utilizing existing climate observation grid data. The quantile mapping method was employed to correct CMIP6 data for the four climate models used^17,18^. This correction aims to enhance the accuracy of future climate factor predictions. Subsequently, we conducted statistical downscaling to achieve a spatial resolution of 1 km. Using a multi-model ensemble composed of four modes, we computed model outputs for various emission scenarios, following the methodology outlined by^19^. In our analyses, we calculated simulated averages for discrete periods: 2021 to 2040 (2030s), 2041 to 2060 (2050s, 2061 to 2080s), and 2081 to 2100 (2090s). These simulated averages were meticulously compared with the ongoing climate state from 1995 to 2020.

#### Land-use data

**Figure 2:**
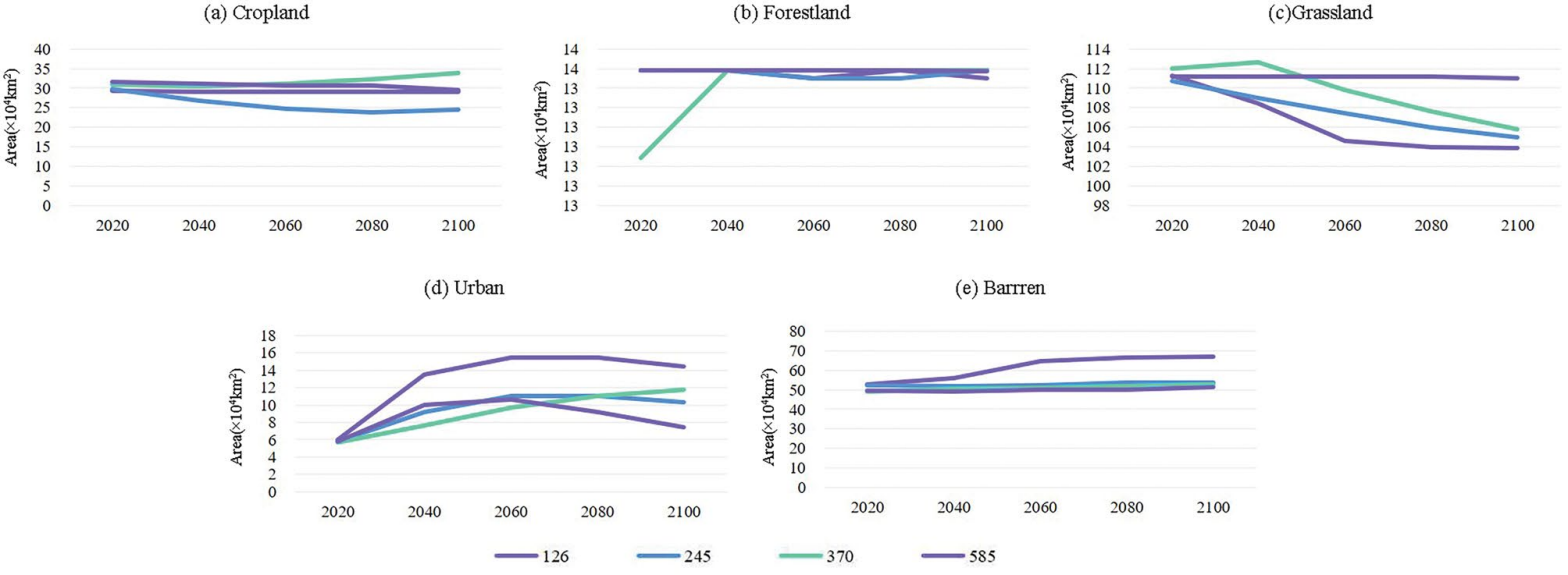
Land Use Demand Trajectories in the Aral Sea basin (Calibrated with 2020 ESA-CCI Land Use Map).

The research utilizes historical land cover data from the latest version of the global land cover dataset provided by the European Space Agency Climate Change Initiative (ESA-CCI). This collection offers data with a spatial resolution of 300 m and covers the temporal span from 1995 to 2020, as detailed in Table 2. To project future land-use trends from 2021 to 2100, researchers rely on data obtained from the Land-Use Harmonization 2 (LUH2) database (http://luh.umd.edu/). LUH2, with a geographical resolution of 0.25°, enables the assessment of annual land-use patterns, potential transitions, and significant agricultural observations from the year 850 to 2100, as documented by^12^. For more detailed forecasts, the land-use map is derived from the 300-m European Space Agency Climate Change Initiative Land Cover (ESA-CCI-LC) dataset, utilizing the methodology described by^20^. In the current investigation, the researchers categorized the 22 land-use types derived from ESA-CCI-LC and the 12 land-use types derived from LUH2 into a comprehensive set of 6 precisely delineated categories. The categorization technique is further detailed in Table S1.The land-use data of LUH2 for 2015 aligns with the historical data acquired from ESA-CCI-LC for the same year. The growth rates for each land-use category were preserved, while considering scenarios that account for the relative changes in LUH2’s 2015 data. Drawing upon the 2015 ESA-CCI-LC land-use map as our baseline, we rigorously outline the projected land-use demands for various domains within the Aral Sea basin. These domains encompass forestland, grassland, barren terrain, cropland, and urban areas, analyzed across four successive timeframes: the 2030s, 2050s, 2070s, and 2090s. These detailed delineations are conducted within the framework of four distinct scenarios (SSP126, SSP245, SSP370, and SSP585), visually represented in Fig. 2, providing substantial evidence for our analysis. Subsequently, all projections pertaining to land use, characterized by a 300 m resolution, undergo meticulous resampling to align with a 1 km scale. This resampling procedure, executed using ArcGIS 10.2, ensures consistency across scales and serves as fundamental input data for the ensuing phase of the Ecological Sensitivity assessment.

#### Soil and socioeconomic data

The fundamental information for our study is derived from the harmonized global soil database (HWSD) at a resolution of 1 km, providing essential soil data. Road network data is acquired from publicly available worldwide road assessment sources. Digital elevation data is obtained from NASA’s Space Shuttle Radar Terrain Mission, with a resolution of 90 m, enabling the derivation of contours, slopes, and aspects throughout the Aral Sea basin (Table 2). To replicate the various factors impacting land growth, a total of nine variables were gathered, including topographical characteristics, population data, and land cover parameters (Table 2). Current climatic conditions are characterized by precipitation and temperature data obtained from the global normal climate dataset provided by WorldClim. This dataset encompasses average values derived from the period between 1970 and 2000. Population data is obtained from the world population plan with a spatial resolution of 1 km. Using ArcGIS software, we employ the Euclidean distance metric to compute the spatial separation between bodies of water and road networks. Subsequently, we resample all environmental data to conform to a resolution of 1 km × 1 km, ensuring compatibility with the land-use simulation model as outlined in Table 2.

### SSPs/RCPs-FLUS-InVEST

In the present study, we employed the FLUS model to simulate land-use changes influenced by human activities and natural factors, as outlined in the work of^21^. To examine the correlation between climate and land-use changes concerning significant ESs within the framework of SSPs-RCPs scenarios, we integrated the FLUS model with the InVEST model. The FLUS model was utilized to generate future land-use estimates by incorporating land demand data generated from the SSPs and RCPs. Subsequently, the land-use projections, along with climate data obtained from GCMs, served as inputs for the InVEST model. The InVEST model was applied to assess modifications in WY, SC, CS, and HQ across historical (1995–2020) and future temporal intervals (2030s, 2050s, 2070s, and 2090s) within the context of SSP scenarios (SSP126, SSP245, SSP370, AND SSP585).

#### Simulation of future land use

The FLUS model is globally recognized for its accurate simulation of diverse land-use scenarios, surpassing conventional models in accuracy and empirical application^21,22^. In this study, we used the FLUS model to simulate future land use under different scenarios using an artificial neural network algorithm. This process involves analyzing foundational land-use data and impact factors to calculate the probability of development for each land-use type. The combination of various factors leads to an overall conversion probability, resembling a roulette competition, which generates simulation outcomes.

##### Adaptive calculations using an artificial neural network

The artificial neural network (ANN) replicates the human brain’s architecture to address complex issues by weighting geographical variables^23,24^. The FLUS model employs this approach to adaptively learn land-use data in the Aral Sea basin, considering factors such as topography, climate, and human activities to predict land type suitability.

##### Roulette-based adaptive inertial competition mechanism

The FLUS model integrates an adaptive competition mechanism based on roulette selection, combining neighborhood weighting and conversion criteria. It organizes land types spatially in future scenarios by adjusting inertia coefficients iteratively to match present and future land demands, significantly enhancing land-use modeling. The formula is as follows:

1 $${TP}_{p,k}^{t}={P}_{p,k}\times {\Omega }_{p,k}^{t}\times {Intertia}_{k}^{t}\times (1-{sc}_{c\to k})$$

2 $${\Omega }_{p,k}^{t}=\frac{{\sum }_{N\times N}con({c}_{p}^{t-1}=k)}{N\times N-1}\times {w}_{k}$$

3 $${Intertia}_{k}^{t}=\left\{\begin{array}{l}{Intertia}_{k}^{t-1}\,\,\,if \left|{D}_{k}^{t-1}\right|\le \left|{D}_{k}^{t-2}\right|\\ {Intertia}_{k}^{t-1}\times \frac{{D}_{k}^{t-2}}{{D}_{k}^{t-1}}\,\,\,if {D}_{k}^{t-1}<{D}_{k}^{t-2}<0\\ {Intertia}_{k}^{t-1}\times \frac{{D}_{k}^{t-1}}{{D}_{k}^{t-2}}\,\,\,if 0<{D}_{k}^{t-2}<{D}_{k}^{t-1}\end{array}\right.$$

The symbol ${TP}_{p,k}^{t}$ denotes the overall probability of transitioning from the original land type in grid *P* to land type *k* at time *t*. The symbol ${\Omega }_{p,k}^{t}$ represents the likelihood of land type *k* occurring in grid *p*, whereas ${\text{Intertia}}_{k}^{t}$ represents the inertia coefficient associated with land type *k* at time *t*. The term *sc*_*c→k*_ is employed to denote the cost linked to the transformation from land type c to land type *k*. The symbol ${\sum }_{N\times N}con({c}_{p}^{t-1}=k)$ denotes the summation of the number of raster cells that are occupied by land type *k* at time *t*−1 inside a moving window of size *N* × *N*. The term wk is used to denote the weight assigned to the interrelations between different land-use categories. In this context, N represents the molar field value inside the cellular automata (CA) framework. The variables ${D}_{k}^{t-1}$ and ${D}_{k}^{t-2}$ denote the disparities between the overall demand at a macro level and the assigned quantity of land type k at time *t*−1 and *t*−2, respectively.

##### Model application and verification

**Figure 3:**
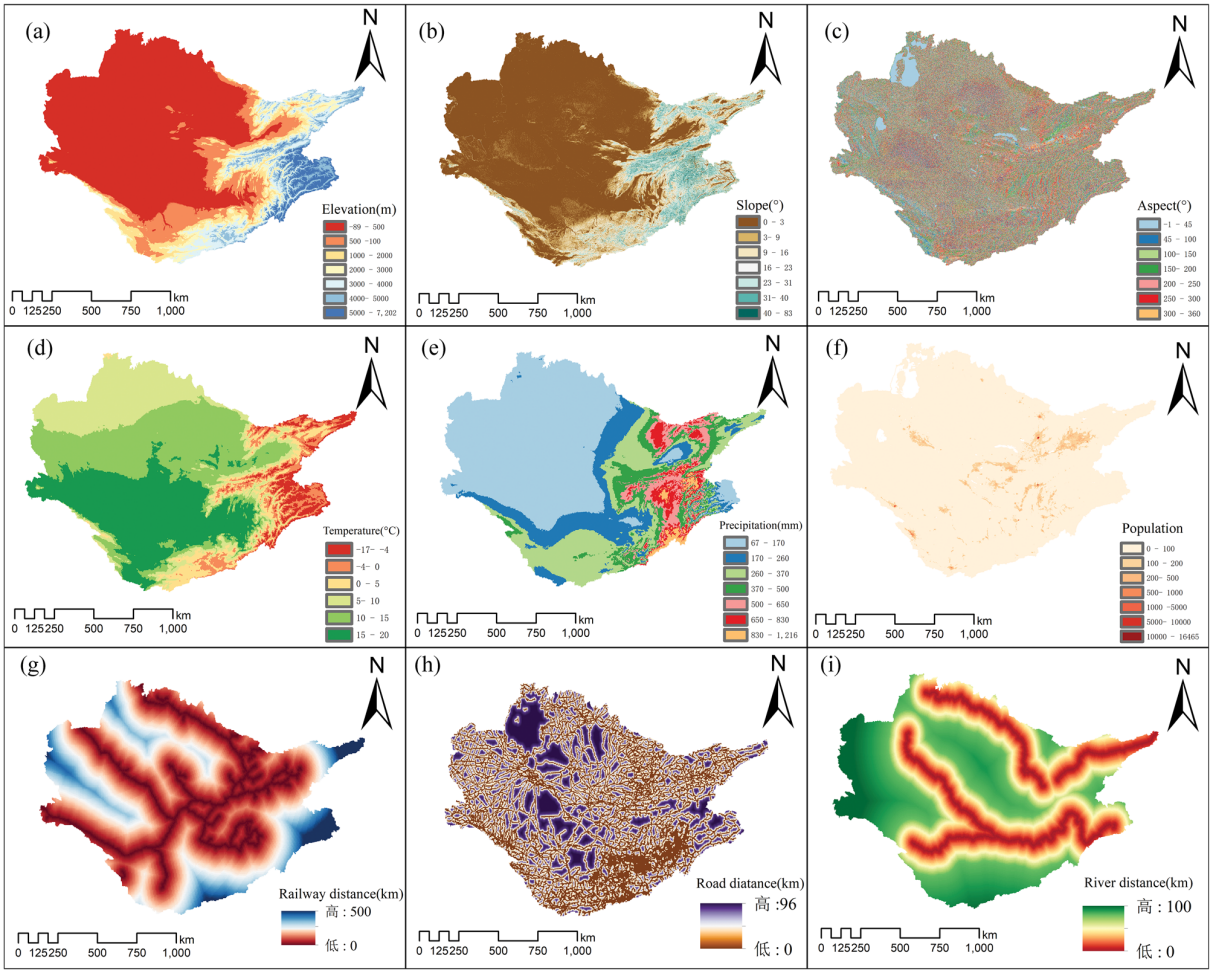
Driving factors affecting land use distribution. Natural forces (**a**–**f**), distance forces (**g**–**i**).

Within the FLUS model framework, we begin by utilizing the 1995 land-use status data from the Aral Sea basin as our initial dataset. This foundational data serves as the basis for subsequent stages, where we import the suitability probability atlas and limiting factors associated with each land type. Concurrently, we establish model parameters critical to the simulation process (Fig. 3). The primary objective of this model is to reproduce the land-use map from 2015, enabling a comprehensive analysis of the generated output in relation to the factual 2015 land-use data. This comparison serves as the basis for evaluating the effectiveness of the FLUS model in simulating real-world scenarios. The evaluation employs two main criteria, namely the kappa coefficient and the FoM, to objectively assess the precision of our land-use models. The mathematical procedure used to compute these measurements may be succinctly explained as follows:

4 $$\text{Kappa}=\frac{{p}_{0}-{p}_{c}}{1-{p}_{c}}$$

The variable ${p}_{0}$ represents the actual accuracy of the simulation, whereas ${p}_{c}$ represents the predicted accuracy of the simulation under random conditions.

5 $$\text{FoM}=B/(\text{A }+\text{ B }+\text{ C }+\text{ D})$$

FoM is a performance index, quantifying the ratio of overlap to total between modeled and actual land-use changes. *A* represents cells truly changed but missed by the simulation, *B* denotes cells correctly changed, *C* is for cells inconsistently changed, and *D* indicates unchanged cells simulated as changed. The FoM, or Figure of Merit, is a numerical measure that spans from 0 to 1. Higher values of FoM indicate more accuracy in Land-Use Change Classification within the modeled region.

#### Assessment of key ESs

The computation of HQ, CS, WY, SC services, and spatial distribution attributes within a representative Aral Sea basin was conducted through the utilization of InVEST models in conjunction with ArcGIS 10.8 software.

##### Water yield

The WY module of the InVEST model applies the water balance principle, utilizing Budyko’s hydrothermal coupled equilibrium hypothesis (1974) and annual average precipitation data. This module considers a wide range of inherent environmental elements, including climatic conditions, terrain, vegetation, soil qualities, and other relevant variables^25^. The formula for the underlying computation is presented as follows:

6 $${Y}_{x,y}=\left(1-\frac{{AET}_{x,j}}{{P}_{x}}\right)\times {P}_{x}$$

The variable ${Y}_{x,y}$ is used to express the water yield of grid x for a certain land-use type *j*. Conversely, the variable ${AET}_{x,j}$ is used to represent the yearly actual evapotranspiration for grid x of the same land-use type *j*. Moreover, the variable ${P}_{x}$ represents the yearly mean precipitation for grid *x*, as elucidated by^26^. Further details and the precise methodology for conducting the calculations can be found in Supplementary Material 1.

##### Soil conservation

The SC assessment in the InVEST model relies on the Universal Soil Loss Equation (USLE). The calculation formula is as follows:

7 $$\Delta USLE=RKLS-USLE=R\times K\times LS\times (1-C\times P)$$

The USLE is a measure of the average annual soil retention expressed in units of metric tonnes per square kilometer per year. It quantifies the amount of soil loss that takes place under plant cover. RKLS denotes the quantification of soil erosion occurring from exposed ground surfaces, whereas LS refers to the combined influence of slope length and slope factor, both of which are derived from the Digital Elevation Model model. The variable *C* represents the dimensionless component associated with vegetation and management elements, whereas the variable *P* represents the dimensionless factor related to SC strategies. The erosion force factor of precipitation runoff, denoted as *R*, is defined in Supplementary Material 1, which provides detailed information on the specific calculation procedures.

##### Carbon storage

The categorization of ecosystem carbon storage is facilitated by the Carbon Storage Module, which classifies it into four main carbon pools: aboveground biocarbon, subsurface biocarbon, soil carbon, and dead organic carbon. A land use-based categorization is utilized to compute and report the mean carbon density for each unique carbon reservoir across diverse land types. Therefore, the overall carbon storage for the designated research region is calculated by multiplying the surface area of each land category by its corresponding carbon density, followed by the summation of these computed values. The procedure for performing calculations is outlined as follows:

8 $${C}_{total}={C}_{above}+{C}_{below}+{C}_{soil}+{C}_{dead}$$

where ${C}_{total}$ represents the total carbon storage within the basin (t/hm^2^), ${C}_{above}$ stands for aboveground carbon storage (t/hm^2^), ${C}_{below}$ represents underground carbon storage (t/hm^2^), ${C}_{soil}$ denotes soil carbon storage (t/hm^2^), and ${C}_{dead}$ signifies dead organic carbon storage (t/hm^2^).

##### Habitat quality

The assessment of habitat quality as a continuous variable is carried out through the Habitat Quality Module, which integrates landscape type sensitivity and the extent of external threats. This evaluation method intricately combines multiple criteria, including the regional impact range of stressors, spatial weighting, the extent of legal land protection, and the influence of both current and changing land cover patterns on habitat quality. The module’s parameters, such as the magnitude of threat factors’ influence, the weighting assigned to these threat factors, the correlation of linear degradation, and the sensitivity of different habitat types to these threats, are established based on relevant research conducted in arid regions^27^ and guidance provided in the InVEST model user manual. The calculation formula is expressed as follows:

9 $${Q}_{xj}={H}_{j}\left(1-\frac{{D}_{xj}^{2}}{{k}^{2}+{D}_{xj}^{2}}\right)$$

where ${Q}_{xj}$ represents the habitat quality index of grid *x* in land use *j*; ${H}_{j}$ signifies the habitat suitability of habitat type *j*, with a value range between 0 and 1; *k* denotes the semi-saturation constant, which is typically set as half of the maximum value of habitat degradation; and *z* stands for a normalized constant, often assigned a value of 2.5^28^.

### Examination of trade-offs and synergies among ecosystem services

A correlation study was conducted to evaluate the trade-offs and synergies among four ESs, namely, water yield, carbon reserves, SC, and habitat quality. The data collection process involved extracting parameters related to ESs from a total of 10,000 randomly generated sites distributed across three distinct watershed units. ArcGIS capabilities, including “Create Random Points” and “Extract Multi Values to Points,” were utilized for this purpose. Subsequently, the “Value Extraction to Point” tool was employed to obtain the respective values of ecosystem service functions. Given the varying magnitudes exhibited by each function, the importance of data standardization became evident. However, the collected data did not adhere to a normal distribution. Therefore, the decision was made to employ the Spearman correlation coefficient to examine the associations between the four ecosystem service functions. The statistical analysis was conducted using SPSS 27.

## Results

### Land-use simulation

**Figure 4:**
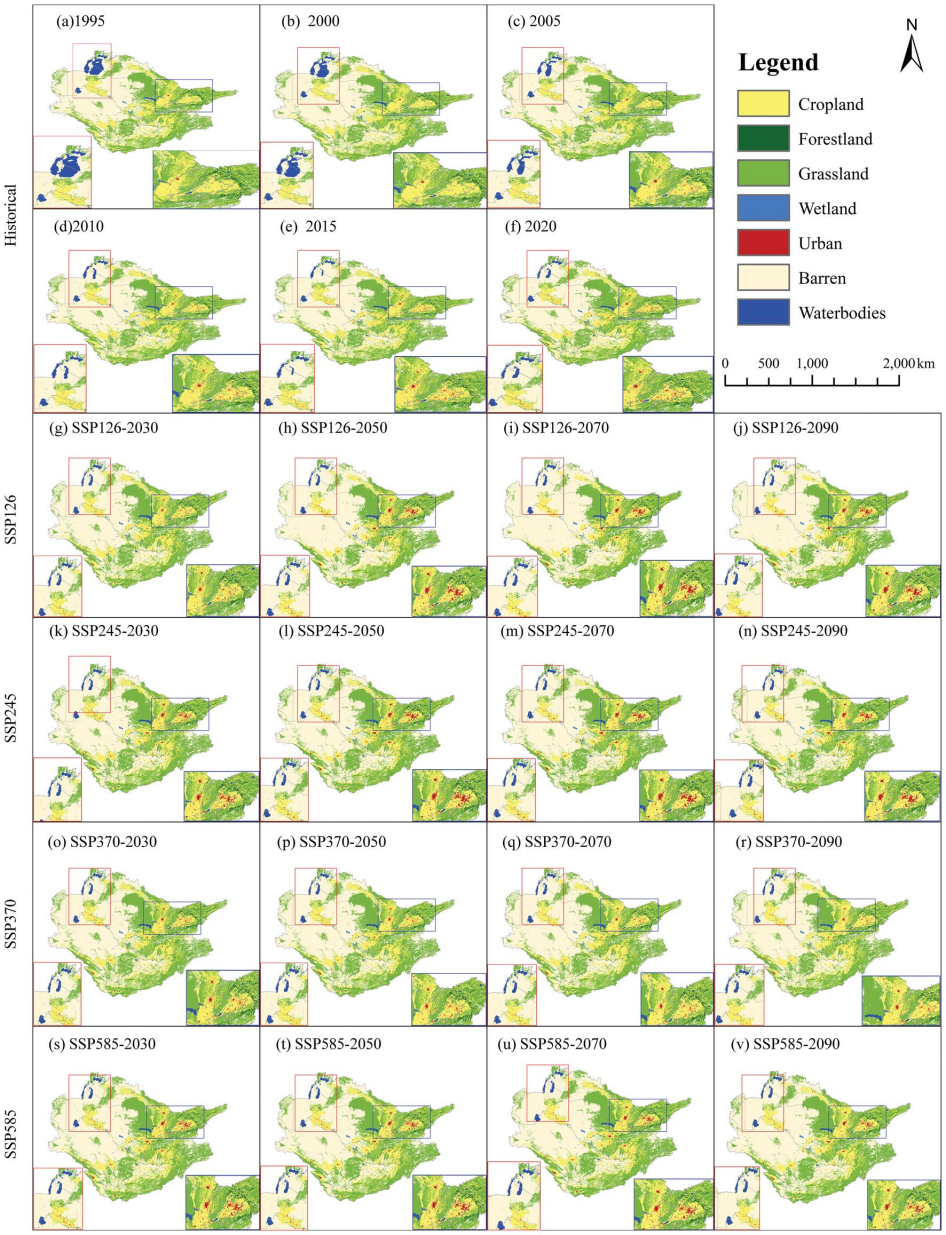
Land-use patterns in Aral sea basin (**a**–**f**) historical period (1995–2020); (**g**–**v**) future four periods (2030s, 2050s, 2070s, and 2090s) under SSP126, SSP245, SSP370, and SSP585.

The evaluation of simulation performance yielded a kappa coefficient of 99.73%, indicating a high level of agreement. Furthermore, the FoM value, standing at 87.09%, underscores the significant accuracy of FLUS, affirming its suitability as a tool for modeling land-use changes in the Aral Sea basin. Upon examining the historical timeframe from 1995 to 2020, as depicted in Fig. 4a–f, a notable surge in urban expansion by 504.65% becomes apparent, accompanied by a substantial decline of 49.59% in the sea surface area within the Aral Sea basin. This phenomenon of urban expansion is primarily concentrated in specific regions, including the capital city and economically advanced metropolitan centers such as Toshkent, Samarkand, and Dushanfe (Fig. 4). Looking ahead to the subsequent four periods (Fig. 4d–o), urban expansion within the Aral Sea basin is expected to persist, with growth rates varying between 116.84 and 134.95% under SSP126, 61.53% and 97.26% under SSP245, 31.83% and 98.85% under SSP370, and 27.09% and 82.38% under SSP585. The ongoing process of urbanization is expected to primarily occur in the capital city and the adjacent Aral Sea region. Across the four scenarios considered, there is expected to be a notable expansion of bare land within the Aral Sea basin, with projected increases ranging from 0.45 to 14.52%. Notably, SSP245 demonstrates the greatest output and the most significant loss, evidenced by a range of agricultural degradation spanning from − 7.32 to 16.73%. In summary, the Aral Sea basin is anticipated to undergo future land-use transformations, characterized by the deterioration of farming (− 0.63% to − 16.73%), degradation of grassland (− 0.18% to − 15.48%), growth of urban areas (27.09% to 134.95%), and an increase in bare land (0.45% to 14.52%).

### Projection of future ESs

#### Water yield

**Figure 5:**
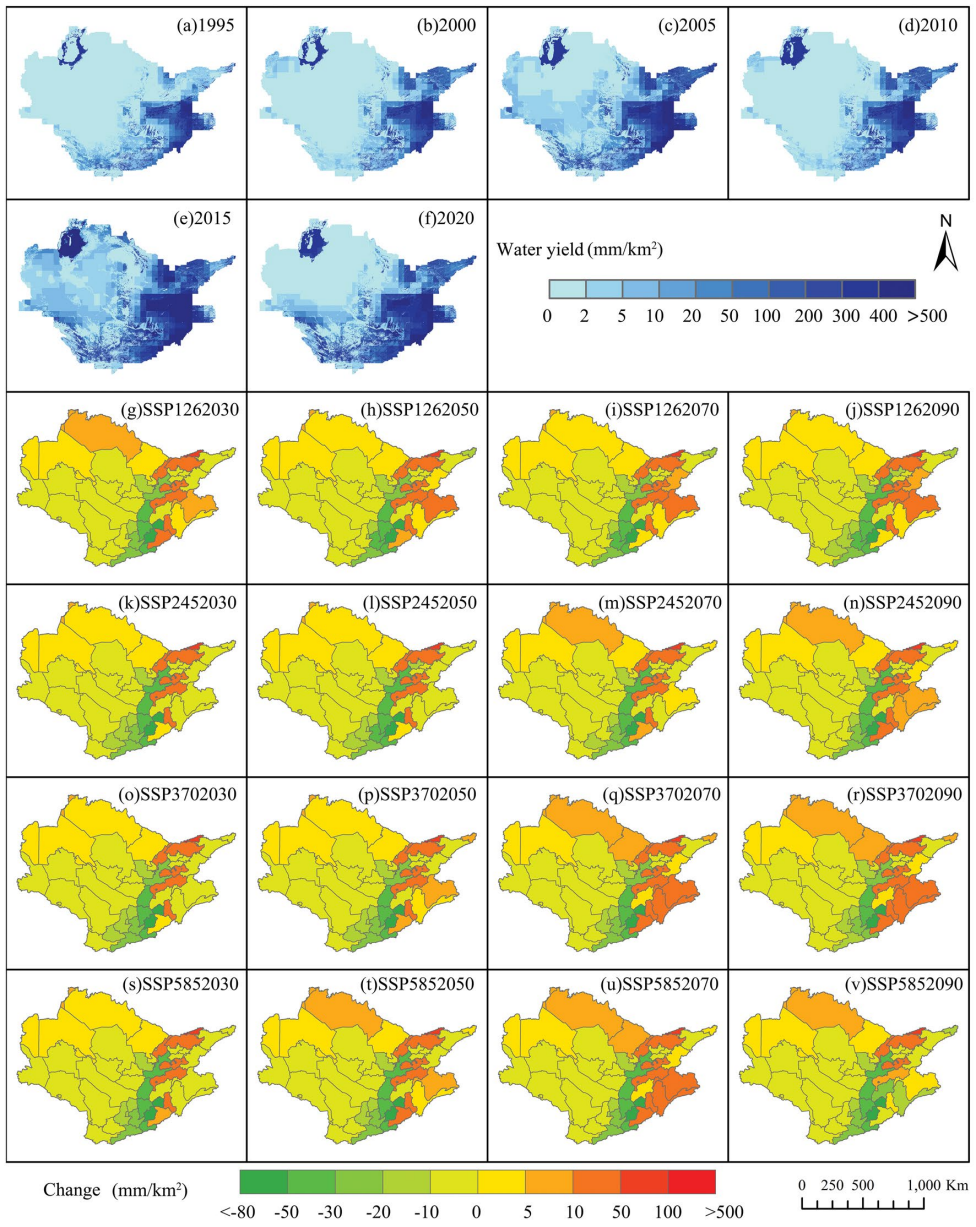
(**a**–**f**) Historical Water Yield (1995–2020). (**g**–**v**) Future Water Yield (2030s, 2050s, 2070s, 2090s) under Different Scenarios.

The total WY of the Aral Sea basin in 1995, 2000, 2005, 2010, 2015, and 2020, measured in average WY depth, was as follows: 2.08 × 10^10^ m^3^ (51.06 mm), 253.89 × 10^8^ m^3^ (48.91 mm), 431.54 × 10^8^ m^3^ (91.66 mm), 284.21 × 108 m^3^ (51.89 mm), 649.73 × 10^8^ m^3^ (95.96 mm), and 463.19 × 10^8^ m^3^ (81.97 mm), respectively. The total water production in the Aral Sea basin experienced a 55.10% increase from 1995 to 2020. Increased WY is observable in the eastern Aral Sea basin as well as in hilly areas. Conversely, lower WY is noted in arid deserts and barren lands. The spatial distribution of water production areas follows a pattern of gradual decrease from the southeast to the northwest. When comparing the predicted WY value of the Aral Sea basin with the historical period of 2020, the WY value is expected to decrease. In four future scenarios, by 2030, compared with 2020, SSP126, SSP245, SSP370, and SSP585 will decrease by 2.03%, 8.11%, 7.64%, and 8.10%, respectively. By 2090, compared with 2020, SSP126, SSP245, SSP370, and SSP585 decreased by 4.94%, 2.00%, 6.02%, and 10.13%, respectively. The annual average values of Afghanistan, Turkmenistan, and Uzbekistan will decrease by 8.62–29.99%, 71.99–78.22%, and 0.04–8.81%, respectively. The annual average WY of Kyrgyzstan and Kazakhstan will increase by 31.30–55.73% and 62.22–76.97%, respectively. Figure 5 shows the chart depicting changes in WY. It provides a clear visual representation of the projected increase in water availability, mostly focused in the mountainous areas of eastern Kazakhstan and the southeastern vicinity of the Aral Sea. The spatial variation map of water is examined, further demonstrating comparable expansion in the eastern Aral Sea basin, southeastern Kyrgyzstan, northern Afghanistan, and southern Kazakhstan. Specifically, increase in WY is noted in provinces such as Badakhshan, Takhar, Baghlan, Jalalabad, Batken, and Gorno-Badakhshan. The regions where a decrease in WY is predicted are mainly distributed in Kunduz, Balkh, Samangan, Jawzjan, Faryab, Sari Pul, Bamyan, Jizzax, and Surxondaryo.

#### Carbon storage

**Figure 6:**
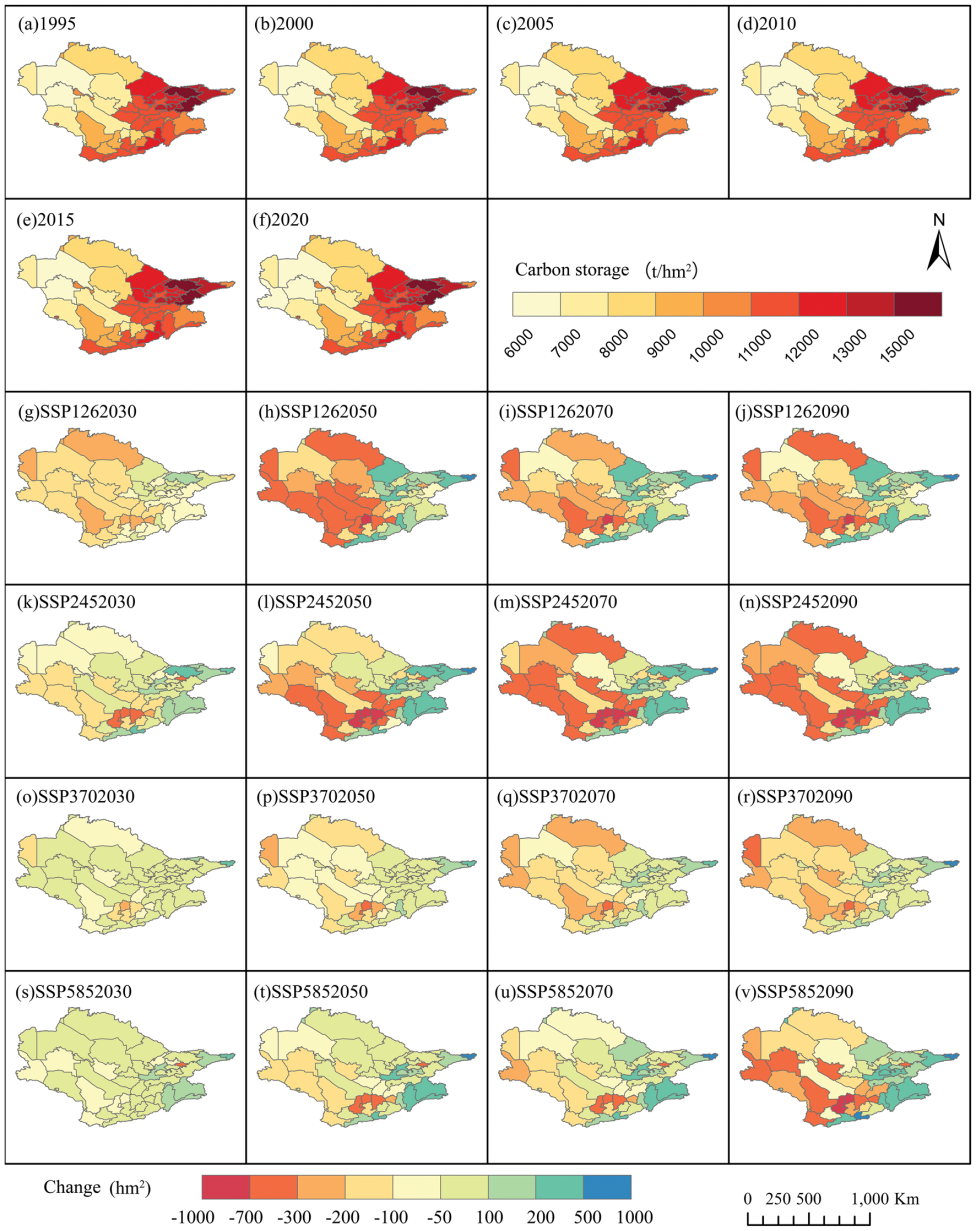
(**a**–**f**) Historical Carbon Storage (1995–2020). (**g**–**v**) Future Carbon Storage (2030s, 2050s, 2070s, 2090s) under Different Scenarios.

The cumulative CS within the Aral Sea basin experienced growth, ascending from 147.62 × 10^8^ t in 1995 to 148.182 × 10^8^ t in 2020. Projected across the future scenarios, the aggregate carbon storage is anticipated to decline from the 2020 levels. In four future scenarios, by 2030, the CS in the Aral Sea basin will continue to decline compared with 2020. The CS in SSP126, SSP245, SSP370, and SSP585 will decrease by 0.08%, 0.57%, 0.41%, and 1.56%, respectively. By 2090, the CS in SSP126, SSP245, SSP370, and SSP585 will decrease by 1.07%, 2.19%, 1.37%, and 5.82% compared with 2020, respectively (Fig. 6). The regions with the most pronounced increase in CS are generally consistent across three scenarios: SSP126, SSP370, and SSP585. These regions are largely situated in the eastern part of the watershed and include Ghor, Bamyan, Naryn, Takhar, Badakhshan, Jalalabad, Gorno-Badakhshan, Sughd, and Issyk-Kul.

#### Habitat quality

**Figure 7:**
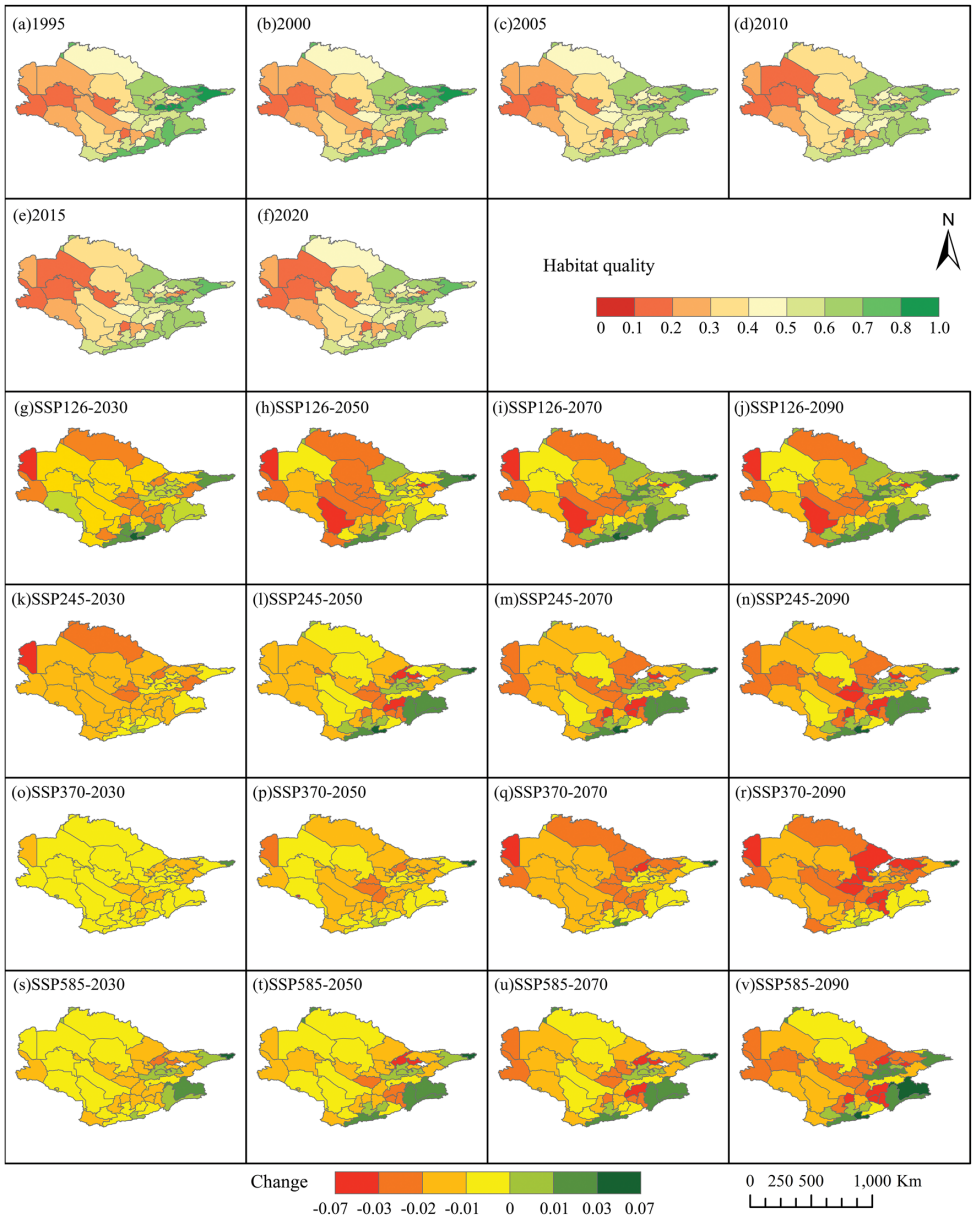
(**a**–**f**) Historical Habitat Quality (1995–2020). (**g**–**v**) Future Habitat Quality (2030s, 2050s, 2070s, 2090s) under Different Scenarios.

The annual average HQ in 1995 and 2020 was 0.412 and 0.406, respectively (Fig. 7a and f); the HQ decreased by 1.5% in 15 years. Under the four future scenarios, by 2030, the HQ of the Aral Sea basin will continue to decline. The HQ in SSP126, SSP245, SSP370, and SSP585 will decline by 3.61%, 1.14%, 1.38%, and 1.24%, respectively. By 2090, compared with 2020, the HQ in SSP126, SSP245, SSP370, and SSP585 will decrease by 10.92%, 2.77%, 5.06%, and 2.09%, respectively. Decline in vegetation cover, coupled with degradation of grasslands and farmland, is a critical factor in the growth and habitat loss of barren landscapes. Growth in urban areas remains a prominent factor in habitat destruction (Fig. 7).

#### Soil conservation

**Figure 8:**
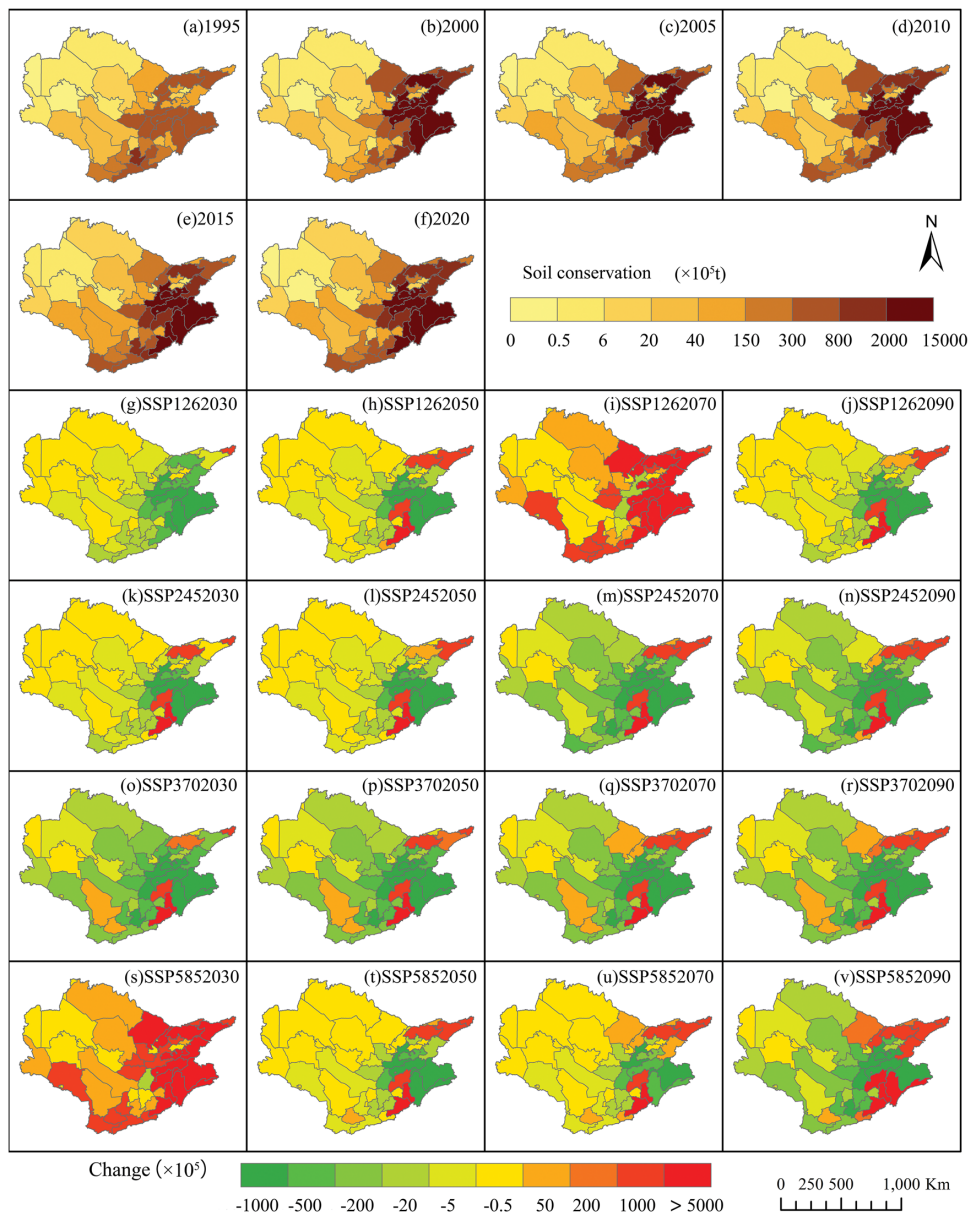
(**a**–**f**) Historical Soil Conservation (1995–2020). (**g**–**v**) Future Soil Conservation (2030s, 2050s, 2070s, 2090s) under Different Scenarios.

The quantities of SC reported in 1995 and 2020 were 4.84 × 108 t and 8.04 × 108 t, respectively. This finding represents a notable increase of 62.81% (Fig. 8a and f). Compared with the predicted values of SC in the Aral Sea basin with the historical period of 2020, the SC value is expected to significantly decrease. In four future scenarios, by 2030, SC in the Aral Sea basin will significantly decrease. The SC in SSP126, SSP245, SSP370, and SSP585 will decrease by 20.55%, 12.65%, 16.14%, and 22.10%, respectively. By 2090, the SC in SSP126, SSP245, SSP370, and SSP585 will decrease by 17.59%, 7.91%, 8.34%, and 1.79%, respectively. A positive trend is noted in SC levels in agriculture, woods, and grasslands, whereas SC in barren land and urban areas declines. From a geographical perspective, the decline in CS efforts is focused on the northern region of the Aral Sea basin. This observation raises concerns about the potential escalation of soil erosion in the mountainous areas of Kyrgyzstan and Tajikistan, situated in the upper Aral Sea basin, in the coming years (Fig. 8).

### Examination of trade-offs and synergies in ecosystem services

Using the geospatial analysis tool ArcGIS alongside the digital elevation map, contour lines at 100-m intervals were extracted within the basin. The 1000-m contour delineated the boundary between mountain pass rivers and alluvial plains, with river dispersion areas consistently lying below the 100-m contour. The 1000 and 100 m contour lines were thus adopted as divisions for the upper, middle, and lower river segments. Alterations in key ESs were then separately assessed for the upstream, midstream, and downstream regions.

**Figure 9:**
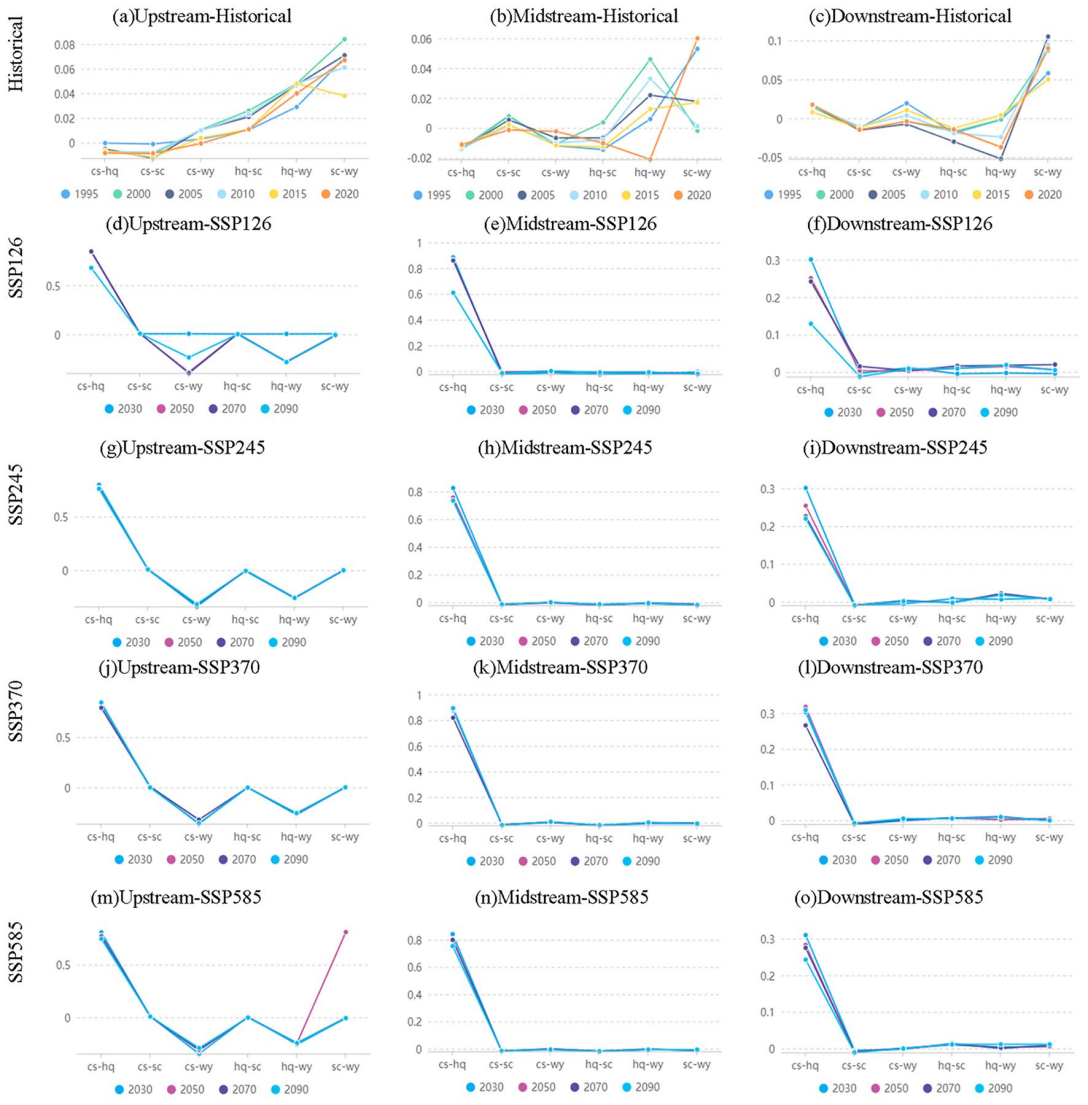
The trade-offs and synergies among four ecosystem services (WY, SC, CS, HQ) in the upper, middle, and lower sectors of the Aral Sea basin are discussed in the supplementary materials.

The examination of spatial correlation has revealed complex interconnections between ESs within the top, middle, and lower regions of the Aral Sea basin, spanning historical periods as well as projected future scenarios. Significant correlations were noted between WY, HQ, and SC in the upper Aral Sea over historical periods (R = 0.29–0.84; *p* < 0.01). Nevertheless, trade-offs occurred between WY and HQ in the forthcoming situations (R =  − 0.283 to − 0.257; *p* < 0.01). In the central region of the Aral Sea, evidence shows a strong positive correlation between historical eras and HQ and WY (R > 0.33; *p* < 0.01). However, the connection between WY and HQ decreased in strength over the four prospective situations. By contrast, the connection between CS and HQ significantly increased, with a correlation coefficient ranging from 0.735 to 0.894 (*p* < 0.01). In the region of the lower Aral Sea, historical SC and WY were positively associated (R = 0.058–0.105; *p* < 0.01). Conversely, an inverse relationship was noted between HQ and WY (R =  − 0.052 to − 0.024; *p* < 0.01). No notable relationships were found among the remaining services. The limited association previously observed between HQ and CS become more evident in the subsequent cases, leading to a positive correlation (R = 0.058–0.105; *p* < 0.01). The strong positive connection previously observed between variable WY and SC decreased in strength (Fig. 9).

## Discussion

### Impact of driving forces on ecosystem services

#### Factors influencing the alteration in water yield services

**Figure 10:**
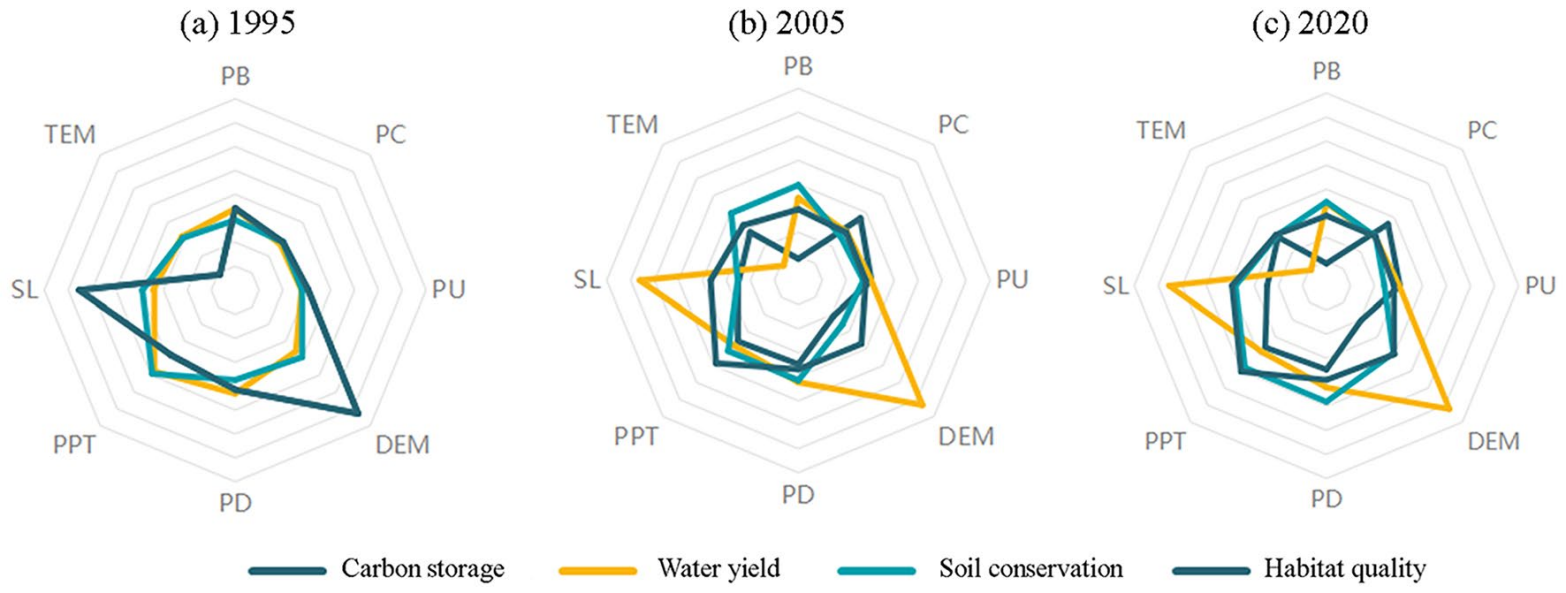
The influence of socio-ecological factors on the four core ecosystem services, including climate variables (PPT and TEM), topography (DEM and SL), vegetation (VC), human population (PD), economic indicators (GDP), land use (PC, PF, PG), and urbanization (PU).

Regarding spatial patterns: The water production capacity displayed a significant negative correlation with altitude (R =  − 0.60; *p* < 0.01), indicating that higher altitudes tend to have lower water production capacity. This relationship is influenced by factors such as precipitation patterns and vegetation distribution, which vary with altitude. In terms of meteorological conditions, there was a clear positive association between water production and precipitation. Precipitation emerged as the primary driver of WY (R = 0.180; *p* < 0.01), consistent with findings from previous studies^29–31^ (Fig. 10). The Aral Sea basin saw a notable 55.98% increase in WY due to heightened precipitation. Additionally, a clear inverse relationship existed between water production capacity and the extent of farmland and urban areas, suggesting reduced capacity in areas with more agriculture and urban development.

#### Factors influencing the carbon storage service

Carbon sequestration is greatly affected by temperature and precipitation, as they play crucial roles in balancing carbon uptake and release. This equilibrium is largely shaped by the input of plant waste and the decomposition of soil organic matter facilitated by microorganisms. Wiesmeier et al.^32^ underscored the role of precipitation in regulating net primary production and its subsequent impact on soil organic matter input. Temperature also significantly influences carbon storage, primarily due to its effect on the microbial breakdown of soil organic matter, which is highly sensitive to temperature changes. According to the study done by^33^, an inverse relationship exists between increasing temperatures and soil organic matter. The decrease in soil organic matter leads to a subsequent reduction in carbon stocks, as shown by the findings of Wiesmeier et al. (2013). In the Aral Sea basin, there is a notable positive correlation between precipitation and CS (R = 0.84; *p* < 0.01), indicating that increased precipitation positively influences carbon storage. Conversely, temperature shows a negative relationship with CS (R =  − 0.209; *p* < 0.01), suggesting that higher temperatures are associated with lower CS levels. The present study unveils a significant finding regarding the correlation between CS and elevation, as depicted in Fig. 10. This finding is consistent with previous research, which has consistently shown a positive link between higher elevations and enhanced carbon sequestration^34,35^.

#### Factors influencing changes in habitat services

Between 1995 and 2020, there was a gradual decline in HQ within the Aral Sea basin, marked by localized degradation. Previous research has identified land use changes as the primary driver of spatial fluctuations in HQ. Improving habitat quality involves recalibrating landscape structure and optimizing layout^36,37^. A significant negative correlation was observed between HQ and the proportion of barren land (R =  − 0.212 to − 0.207; *p* < 0.01), indicating that increased barren land corresponds to habitat deterioration. Altitude emerged as a key influencer of habitat quality, particularly in the upper Aral Sea region, which exhibited favorable habitat conditions. Additionally, higher HQ was observed in the southeast compared to the relatively lower quality in the northwest. This spatial pattern aligns with the distribution of altitude, highlighting the substantial explanatory power of terrain attributes over habitat quality diversity, consistent with the findings of^38^. Our projections indicate that increased barren land and urban expansion will lead to a decline in HQ across the Aral Sea basin (3.61–10.92%), with the SSP126 scenario showing the most significant decline (Fig. 7).

#### Factors influencing the evolution of soil conservation services

The research findings indicate that SC services are significantly influenced by the synergistic interaction of climate change, particularly rainfall patterns, and alterations in land cover. Topographic conditions emerge as the primary spatial drivers, consistent with previous studies^39^. Soil erosion tends to occur in regions characterized by steep terrain, high precipitation, and changes in runoff patterns. Steeper slopes amplify runoff, leading to the transport of sediment, while soil erosion resistance decreases with increasing gradient^40^. Projections indicate increased soil erosion risks in the upper mountainous regions of the Aral Sea. Effective strategies to address this issue include expanding forest coverage and implementing appropriate management practices in these elevated areas.

### Strategies to improve ecosystem services in the Aral Sea basin

Achieving harmony between humans and nature is crucial for the sustainability of ESs, necessitating a balance between supply and demand as human needs expand. In areas experiencing declines in ecosystem service supply, governmental interventions such as ecological restoration, pollution reduction, and enhanced nature reserves can be implemented. Notably, initiatives like China’s Three North Shelter Forest System Project, Natural Forest Conservation Program, and “Grain for Green Program” have notably bolstered ecosystem service supply, particularly in northern China^41,42^. The study highlights that water-producing areas in the Aral Sea basin primarily originate from upstream mountainous regions in countries like Tajikistan and Kyrgyzstan, while downstream countries such as Turkmenistan, Kazakhstan, and Uzbekistan rely heavily on agricultural lands. This spatial and temporal mismatch between water resources and agricultural land exacerbates complexities in water resource allocation. It is recommended to establish comprehensive water resource management mechanisms aimed at reducing the disparity in water resource sharing between upstream and downstream countries, alongside the adoption of efficient agricultural irrigation methods like drip irrigation to minimize water waste and enhance management efficiency. Additionally, fostering cooperation among Aral Sea basin countries on transboundary river management is essential for promoting sustainable development and resource preservation^43,44^.

### Model comparison strengths and uncertainties

The InVEST model is widely acknowledged as a valuable tool for conducting comprehensive assessments of ESs^9,45^. Choi, Hyun-Ah^46^ analyzed 11 water supply models (AIM, ATEAM, CENTURY, SWAT, GUMBO, InVEST, PLM, SAVANNA, WaSSI, WaterGAP, WBM), assessing their usability for quantifying water supply services. Their findings highlight the suitability of the InVEST and WaterGAP models for this purpose. Feng Juan^47^ demonstrated that the InVEST model exhibits higher simulation accuracy compared to the SWAT model, particularly on an annual scale. Therefore, this study selected the InVEST model to analyze the spatial distribution of water production capacity in the Aral Sea basin. Land-use simulation models serve as efficient and reproducible tools for examining the causes and consequences of future changes in landscape dynamics influenced by socioeconomic and natural environmental factors. Among various geographical simulation models such as cellular automata^48^, CLUE-S model^49^, and FLUS model^50^ have been widely used in land-use simulation research. The FLUS model stands out for its introduction of an adaptive inertia competition mechanism based on roulette selection, improving upon the traditional cellular automaton model. Comparative studies have demonstrated the FLUS model’s superiority over others like CLUE-S and ANN-CA in terms of simulation accuracy. Widely employed in land-use simulation research, the FLUS model aids researchers and policymakers in formulating suitable strategies to adapt to the natural environment amid global climate changes^51,52^.

However, recognizing the inherent limitations of this approach is essential. Specifically, the accuracy of predictions generated by the InVEST water production model may be compromised in areas characterized by basal flows, extensive snow cover, or shallow soils. Previous research by Dennedy-Frank et al. (2016) has demonstrated reduced accuracy of the InVEST water model in regions with significant baseflow, extensive snow accumulation, or shallow soil conditions. Furthermore, water-related modeling techniques and ecosystem service indicators are inherently more complex than those used for carbon analysis. This complexity can pose challenges when comparing results. Despite these constraints, our research sheds light on the dynamic changes in land-use patterns and essential ESs within the Aral Sea basin. The findings presented in this study provide a robust scientific foundation for effectively addressing future climatic shifts and make a significant contribution to aligning socioeconomic development with the preservation of ecological and environmental integrity.

## Conclusion

In our research, we introduce an innovative integrated framework merging the FLUS and InVEST models to simulate land-use dynamics and ecosystem service provision in the Aral Sea basin. This framework incorporates SSP-RCP scenarios from CMIP6 to capture potential future changes. Our primary findings can be summarized as follows: Continuous decline in agriculture, forest land, and grassland, juxtaposed with ongoing expansion of bare land and urban areas, highlights the escalating conflict between agricultural and urban growth over time. Temporal and regional changes in ESs reveal a notable decline across the Aral Sea basin, especially evident in the SSP245 scenario. The extension of land categories unsuitable for supporting habitats, such as areas designated for building, poses a significant threat to high-quality habitat areas like woods, water bodies, and grasslands, intensifying habitat quality deterioration. Correlation analysis underscores trade-offs between carbon storage and habitat quality, as well as water yield, along with synergistic linkages between SC and CS, WY and HQ, and WY and SC. Future projections anticipate a strengthened link between HQ and SC, whereas the relationship between HQ, WY, and SC is expected to weaken. Examination of drivers reveals a significant correlation between natural factors and WY, SC, and CS. Conversely, HQ exhibits higher vulnerability to disruptions caused by human activities. Thus, stakeholders and policymakers must devise tailored management plans for land resources that account for the unique factors influencing corresponding ESs.

### Supplementary Information

Supplementary Information.

## Data Availability

The datasets generated during the current study are available from the corresponding author on reasonable request.
