# Supplementary material for: Spatiotemporal variations of ecosystem services in the Aral Sea basin under different CMIP6 projections
Source: Sci Rep. 2024 May 28;14:12237. doi: 10.1038/s41598-024-62802-9 (PMC11133489; doi:10.1038/s41598-024-62802-9)
Supplement: Supplementary file 1 — Supplementary Information. [file 41598_2024_62802_MOESM1_ESM.pdf]

### 2.3.2.1 Water yield

The formula for calculation is as follows:

$$Y_{xj} = (1 - \frac{AET_x}{P_x}) \times P_x$$

$$\frac{AET_x}{P_x} = \frac{1 + w_x R_x}{1 + w_x R_{xj} \ + \frac{1}{R_x}}$$

$$w_x = Z \times ({}^{AWC_x}/_{P_x})$$

$$R_x = \frac{k_x \times ET_{ox}}{P_x}$$

$$AWC_x = Min(MSD_x, RD_x) \times PAWC_x$$

Where $Y_{xj}$ is the annual average water production of grid *x*; Since the actual annual evapotranspiration cannot be measured directly, the curve can be used to approximate the $AWC_x/P_x$ calculation. $R_x$ value is dimensionless, which is the dryness index of grid *x*, and can be calculated from potential evapotranspiration and rainfall; $w_x$ is an empirical parameter used to describe climate soil properties, which can be calculated by the available water content of vegetation and annual rainfall. $AWC_x$ is the available water content of vegetation, which is used to determine the total water stored and provided by soil for plant growth due to soil texture and effective soil depth. *Z* is called Zhang coefficient, which is an empirical constant and represents the parameters of seasonal rainfall distribution and rainfall depth. The $Z$ value is close to 10 for areas with mainly rainfall in winter, and close to 1 for humid areas with evenly distributed rainfall and areas with mainly rainfall in summer; $ET_{0x}$ is the potential evapotranspiration in grid *x*, which reflects the evapotranspiration capacity determined under weather and climate conditions. The InVEST model uses the Modified-Hargreaves method to calculate the potential evapotranspiration; $k_x$represents the evapotranspiration coefficient of vegetation or crops, and different land use types have different evapotranspiration coefficients; $MSD_x$ (Max Soil Depthx) is the maximum soil depth; $RD_x$ is the root depth; and represents the available moisture content of plants.

### 2.3.2.2 Soil conservation

The model calculation formula is as follows:

$$SEDRET_x = RKLS_x - USLE_x + SEDR_x$$

$$RKLS_x = R_x \times K_x \times LS_x$$

$$USLE_x = R_x \times K_x \times LS_x \times P_x \times C_x$$

$$SEDR_x = SE_x \sum_{y=1}^{x-1} USLE_y \prod_{z=y+1}^{x-1} (1 - SE_x)$$

Formula: $SEDRET_x$, $RKLS_x$, $USLE_x$, $SEDR_x$, and $USLE_y$ are the soil retention amount of the grid *x*, respectively potential soil erosion, actual erosion after considering management and engineering measures, sediment retention, and actual erosion on the uphill grid *y* after considering management and engineering measures,

all are in *t*. $R_x$, $K_x$, $LS_x$, $C_x$, and $P_x$ are the precipitation erosion factors, soil erosion factors, slope length factor, vegetation cover management factor and soil conservation separation measures factor of grid *x*, respectively.

$$R = \sum_{i=1}^{12} \left\{ 1.735 \times 10^{\left(1.5 \log \frac{P_i^2}{P} - 0.08188\right)} \right\}$$

$$K = 0.1317 \times \{0.2 + 0.3 \times exp[-0.0256SAN(1 - {}^{SIL}/_{100})]\} \left(\frac{SIL}{CLA + SIL}\right)^{0.3}$$

$$\times \left[1 - 0.25 \times \frac{O}{O + \exp{(3.72 - 2.95O)}}\right] \times \left[1 - 0.7 \times \frac{SN_1}{SN_1 + \exp{(22.9SN_1 - 5.51)}}\right]$$

$$SN_1 = 1 - \frac{SAN}{100}$$

$$LS = \frac{\left(A_{i,j-in} + D^2\right)^{m+1} - A_{i,i-in}^{m+1}}{D^{m+2} \times x_{i,j}^m \times 22.13^m}$$

$$m = \frac{\beta}{(1 + \beta)}$$

$$\beta = \frac{(\sin\theta\,/0.0896)}{3 \times \sin\theta^{0.8} + 0.56}$$

$$C = exp\left[-x \times \frac{NDVI}{(y - NDVI)}\right]$$

where, $K$ refers to the soil erodibility factor (t·ha·h/ha·MJ·mm); $R$ is to the rainfall erosivity factor (MJ·mm/ha·h·a); $LS$ refers to the topographic factor, including steepness and slope length; $C$ is the vegetation cover practice factor; and *P* is the soil measures factor. $P_j$ is the annual total precipitation (mm). $P_i$ is the monthly precipitation (mm); *O*, *CLA*, *SAN*, *and SIL* refers to the percent of organic matter (%),clay (%), sand (%) and silt (%), respectively; $\theta$ presents angle of slope; *x* and *y* are the parameters controling the shape of the NDVI curve, Knijff et al. (2000)' research have demonstrated that $x = 2$ and $y = 1$ can provide reliable results

**Table S1. Reclassification for land use simulations in this study.**

| LUH2 land classes | ESA-CCI land classes | LULC |
|---|---|---|
| C3 annual crop | Cropland, rainfed | Cropland |
| C3 perennial crop | Herbaceous cover | |
| C4 annual crop | Tree or shrub cover | |
| C4 perennial crop | Cropland, irrigated or post-flooding | |
| C3 nitrogen-fixing crop | Mosaic cropland (>50%)/natural vegetation (tree, shrub, herbaceous cover) (<50%) | |
| | Mosaic natural vegetation (tree, shrub, herbaceous cover) (>50%)/cropland (<50%) | |
| Forested primary Land | Tree cover, broadleaved, evergreen, closed to open (>15%) | Forestland |
| | Tree cover, broadleaved, deciduous, closed to open (>15%) | |
| Potentially forested secondary land | Tree cover, broadleaved, deciduous, closed (>40%) | |
| | Tree cover, broadleaved, deciduous, open (15–40%) | |
| | Tree cover, needleleaved, evergreen, closed to open (>15%) | |
| | Tree cover, needleleaved, evergreen, closed (>40%) | |
| | Tree cover, needleleaved, evergreen, open (15–40%) | |
| | Tree cover, needleleaved, deciduous, closed to open (>15%) | |
| | Tree cover, needleleaved, deciduous, closed (>40%) | |
| | Tree cover, needleleaved, deciduous, open (15–40%) | |
| | Mosaic tree and shrub (>50%)/herbaceous cover (<50%) | |
| | Mosaic herbaceous cover (>50%)/tree and shrub (<50%) | |
| Managed pasture | Shrubland | Grassland |
| Rangeland | Evergreen shrubland | |
| | Deciduous shrubland | |
| | Grassland | |
| | Lichens and mosses | |
| | Sparse vegetation (tree, shrub, herbaceous cover) (<15%) | |
| | Sparse tree (<15%) | |
| | Sparse shrub (<15%) | |
| | Sparse herbaceous cover (<15%) | |
| | Shrub or herbaceous cover, flooded, fresh/saline/brakish water | |
| Urban land | Urban areas | Urban |
| Non-forested primary land | Bare areas | Barren |
| | Consolidated bare areas | |
| Potentially non-forested secondary land | Unconsolidated bare areas | |
| None | Water bodies | Water |
| | Tree cover, flooded, fresh or brakish water | |
| | Tree cover, flooded, saline water snow and ice | |

**Table S2. The neighborhood weights for individual land use type.**

| | Cropland | Forestland | Grassland | Water | Barren | Urban |
|---|---|---|---|---|---|---|
| Weight | 0.5 | 1 | 0.3 | 0.9 | 0.5 | 1 |

**Table S3. Cost Matrix of land use pairs.**

| Land use types | Cropland | Forestland | Grassland | Water | Barren | Urban |
|---|---|---|---|---|---|---|
| Cropland | 1 | 1 | 0 | 0 | 1 | 1 |
| Forestland | 1 | 1 | 0 | 0 | 1 | 1 |
| Grassland | 1 | 1 | 1 | 0 | 0 | 1 |
| Water | 0 | 0 | 0 | 1 | 1 | 0 |
| Barren | 1 | 1 | 1 | 0 | 0 | 1 |
| Urban | 1 | 1 | 1 | 0 | 1 | 1 |

**Table S4. The C and P factor values of different land use types**

| Land use types | C | P |
|---|---|---|
| Cropland | 0.20 | 0.15 |
| Forestland | 0.05 | 1 |
| Grassland | 0.3 | 1 |
| Water | 0 | 0 |
| Barren | 0 | 1 |
| Urban | 1 | 1 |

**Table S5. Carbon density in different land use types (kg C/m$^2$)**

| Land use types | C_above | C_below | C_soil | C_dead |
|---|---|---|---|---|
| Cropland | 6.4 | 17.92 | 88.5 | 2.13 |
| Forestland | 89.5 | 21.48 | 148.7 | 18.8 |
| Grassland | 8.1 | 22.68 | 86.7 | 2.7 |
| Wetland | 4.01 | 0 | 146 | 0 |
| Urban | 14.3 | 22.88 | 60 | 2 |
| Barrren | 4 | 6.4 | 33.5 | 2 |
| Water | 1.31 | 2.42 | 29.9 | 0.35 |

**Table S6. The weight and the maximum influence distance of the threat source.**

| Threat factor | Distance/km | Weight | Decay Type |
|---|---|---|---|
| Urban | 8 | 1 | exponential |
| Bareland | 3 | 0.4 | linear |
| Cropland | 2 | 0.2 | linear |
| Road | 2 | 0.7 | exponential |

**Table S7. Sensitivity of land used type to habitat threat factors.**

| Land use | Habitat suitability | L_urban | L_bareland | L_cropland | L_road |
|---|---|---|---|---|---|
| Cropland | 0.3 | 0.9 | 0.5 | 0.2 | 0 |

| | | | | | |
|---|---|---|---|---|---|
| Forestland | 1 | 0.8 | 0.2 | 0.5 | 0.3 |
| Grassland | 0.9 | 0.5 | 0.3 | 0.2 | 0.3 |
| Wetland | 0.7 | 0.5 | 0.4 | 0.3 | 0.7 |
| Urban | 0 | 0 | 0 | 0 | 0.6 |
| Barrren | 0.1 | 0.3 | 0.2 | 0.1 | 0 |
| Water | 1 | 0.6 | 0.5 | 0.4 | 0 |

**Table S8. Tradeoffs and synergies among four ecosystem services (WY: water yield; SC: soil conservation; CS: Carbon storage; HQ: Habitat quality) in the Upstream,middle stream and Downstream in Aral Sea Basin under historical (His) and future scenarios. **$p < 0.01$; * $p < 0.05$**

| Upstream | 1995 | 2000 | 2005 | 2010 | 2015 | 2020 | SPP1262030 | SSP1262050 | SSP1262070 | 1262090SSP | SSP2452030 |
|---|---|---|---|---|---|---|---|---|---|---|---|
| **CS-HQ** | -0.0003 | -0.0063 | -0.0052 | -0.0073 | -0.0065 | -0.0084 | .844** | .843** | .845** | .678** | .799** |
| **CS-SC** | -0.0011 | -0.0092 | -0.0126 | -0.0077 | -0.0116 | -0.0087 | 0.0061 | 0.0071 | 0.0072 | 0.0080 | 0.0080 |
| **CS-WY** | 0.0030 | 0.0105 | 0.0104 | 0.0099 | 0.0038 | -0.0007 | 0.0064 | -.384** | -.395** | -.235** | -.340** |
| **HQ-SC** | 0.0103 | .026** | .021* | .023* | 0.0108 | 0.0109 | 0.0038 | 0.0068 | 0.0070 | 0.0020 | 0.0004 |
| **HQ-WY** | .029** | .048** | .047** | .048** | .048** | .040** | 0.0053 | -.277** | -.283** | -.282** | -.257** |
| **SC-WY** | .069** | .084** | .071** | .061** | .038** | .067** | 0.0071 | -0.0055 | -0.0117 | -0.0065 | -0.0011 |
| **Upstream** | **SSP2452050** | **SSP2452070** | **SSP2452090** | **SSP3702030** | **SSP3702050** | **SSP3702070** | **SSP3702090** | **SSP5852030** | **SSP5852050** | **SSP5852070** | **SSP5852090** |
| **CS-HQ** | .768** | .766** | .763** | .827** | .837** | .791** | .844** | .809** | .776** | .772** | .748** |
| **CS-SC** | 0.0073 | 0.0075 | 0.0074 | 0.0063 | 0.0029 | 0.0070 | -0.0006 | 0.0087 | 0.0072 | 0.0085 | 0.0079 |
| **CS-WY** | -.320** | -.322** | -.318** | -.362** | -.359** | -.321** | -.357** | -.346** | -.313** | -.309** | -.292** |
| **HQ-SC** | -0.0059 | -0.0066 | -0.0063 | -0.0002 | 0.0005 | -0.0016 | -0.0024 | 0.0040 | 0.0003 | 0.0013 | -0.0025 |
| **HQ-WY** | -.259** | -.262** | -.259** | -.271** | -.262** | -.256** | -.256** | -.258** | -.245** | -.243** | -.244** |
| **SC-WY** | 0.0002 | -0.0003 | -0.0005 | 0.0026 | 0.0019 | 0.0003 | 0.0007 | -0.0068 | 0.8119 | -0.0041 | -0.0070 |
| **Midstream** | **1995** | **2000** | **2005** | **2010** | **2015** | **2020** | **SSP1262030** | **SSP1262050** | **SSP1262070** | **SSP1262090** | **SSP2452030** |
| **CS-HQ** | -0.01357696 | -0.01446408 | -0.01389252 | -0.013731866 | -0.012223852 | -0.011095145 | .885** | .863** | .860** | .609** | .826** |
| **CS-SC** | 0.0081 | 0.0079 | 0.0054 | 0.0017 | 0.0023 | -0.0013 | -.024* | -0.0053 | -0.0104 | -0.0151 | -0.0152 |
| **CS-WY** | -0.0119 | -0.0108 | -0.0067 | -0.0101 | -0.0117 | -0.0022 | -0.0118 | -0.0021 | -0.0009 | 0.0009 | -0.0009 |
| **HQ-SC** | -0.0145 | 0.0038 | -0.0066 | -0.0079 | -0.0126 | -0.0102 | -.024* | -0.0074 | -0.0091 | -0.0096 | -.022* |
| **HQ-WY** | 0.0060 | .046** | .022* | .033** | 0.0126 | -.021* | -0.0193 | -0.0093 | -0.0084 | -0.0083 | -0.0029 |
| **SC-WY** | .053** | -0.001801043 | 0.017725285 | 0.001169106 | 0.017095813 | .060** | -0.006172083 | -0.017447343 | -.023* | -.021* | -0.0118 |
| **Midstream** | **SSP2452050** | **SSP2452070** | **SSP2452090** | **SSP3702030** | **SSP3702050** | **SSP3702070** | **SSP3702090** | **SSP5852030** | **SSP5852050** | **SSP5852070** | **SSP5852090** |
| **CS-HQ** | .757** | .735** | .735** | .876** | .887** | .819** | .894** | .842** | .804** | .799** | .754** |
| **CS-SC** | -0.0161 | -0.0152 | -0.015162585 | -0.0148 | -0.0136 | -0.0144 | -0.0173 | -0.0162 | -0.0157 | -0.0157 | -0.0150 |
| **CS-WY** | -0.0038 | 0.0000 | 0.000371934 | 0.0030 | 0.0059 | 0.0067 | 0.0088 | -0.0011 | -0.0004 | -0.0017 | -0.0058 |

| | | | | | | | | | | | |
|---|---|---|---|---|---|---|---|---|---|---|---|
| **HQ-SC** | -0.0175 | -0.0151 | -0.014761797 | -0.018851777 | -.020* | -.020* | -0.018300747 | -0.018195998 | -0.018834228 | -0.017675589 | -0.018549616 |
| **HQ-WY** | -0.0055 | -0.0076 | -0.005475314 | -0.0046 | 0.0017 | -0.0006 | 0.0027 | -0.0013 | -0.0029 | -0.0043 | -0.0061 |
| **SC-WY** | -0.0133 | -0.0196 | -0.019105952 | -0.0059 | -0.0001 | -0.0014 | -0.0061 | -0.0148 | -0.0088 | -0.0085 | -0.0057 |
| | | | | | | | | | | | |
| **Downstream** | **1995** | **2000** | **2005** | **2010** | **2015** | **2020** | **SSP1262030** | **SSP1262050** | **SSP1262070** | **SSP1262090** | **SSP2452030** |
| **CS-HQ** | 0.0173 | 0.0148 | 0.0167 | 0.0162 | 0.0079 | 0.0176 | .301** | .251** | .242** | .129** | .301** |
| **CS-SC** | -0.0125 | -0.0150 | -0.0154 | -0.0106 | -0.0125 | -0.0146 | -0.0039 | 0.0034 | 0.0152 | -0.0124 | -0.0079 |
| **CS-WY** | 0.0193 | -0.0038 | -0.0075 | 0.0036 | 0.0104 | -0.0039 | 0.0115 | 0.0031 | 0.0039 | 0.0086 | -0.0055 |
| **HQ-SC** | -0.0189 | -0.0170 | -.030** | -0.0189 | -0.0129 | -0.0143 | -0.0046 | 0.0102 | 0.0164 | 0.0093 | 0.0082 |
| **HQ-WY** | -0.001853875 | -0.00155277 | -.052** | -.024* | 0.004017139 | -.037** | -0.002568677 | 0.014480654 | 0.018449714 | 0.01889104 | 0.006579179 |
| **SC-WY** | .058** | .087** | .105** | .094** | .050** | .090** | -0.0042 | 0.0064 | .020* | 0.0051 | 0.0095 |
| **Downstream** | **SSP2452050** | **SSP2452070** | **SSP2452090** | **SSP3702030** | **SSP3702050** | **SSP3702070** | **SSP3702090** | **SSP5852030** | **SSP5852050** | **SSP5852070** | **SSP5852090** |
| **CS-HQ** | .254** | .226** | .220** | .303** | .318** | .266** | .309** | .310** | .283** | .275** | .243** |
| **CS-SC** | -0.0096 | -0.0090 | -0.009532524 | -0.0107 | -0.0079 | -0.0108 | -0.0076 | -0.0050 | -0.0079 | -0.0089 | -0.0109 |
| **CS-WY** | 0.0024 | 0.0033 | 0.000824661 | -0.001008719 | 0.001820605 | 0.001850075 | 0.004840616 | -0.001946927 | -0.000388703 | 0.001115612 | 5.45728E-06 |
| **HQ-SC** | -0.0010 | -0.0025 | -0.002243046 | 0.0081 | 0.0063 | 0.0066 | 0.0055 | 0.0144 | 0.0104 | 0.0110 | 0.0114 |
| **HQ-WY** | .021* | .022* | 0.017561017 | 0.0020 | 0.0022 | 0.0105 | 0.0092 | -0.0005 | 0.0036 | 0.0026 | 0.0117 |
| **SC-WY** | 0.0074 | 0.0073 | 0.006842066 | 0.0052 | 0.0048 | -0.0008 | -0.0001 | 0.0100 | 0.0052 | 0.0068 | 0.0116 |
